# A Multi-screening Evaluation of the Nutritional and Nutraceutical Potential of the Mediterranean Jellyfish *Pelagia noctiluca*

**DOI:** 10.3390/md17030172

**Published:** 2019-03-17

**Authors:** Rosaria Costa, Gioele Capillo, Ambrogina Albergamo, Rosalia Li Volsi, Giovanni Bartolomeo, Giuseppe Bua, Antonio Ferracane, Serena Savoca, Teresa Gervasi, Rossana Rando, Giacomo Dugo, Nunziacarla Spanò

**Affiliations:** 1 Dipartimento di Scienze Biomediche, Odontoiatriche, e delle Immagini Morfologiche e Funzionali (Biomorf), University of Messina, Viale Annunziata, 98168 Messina, Italy; costar@unime.it (R.C.); gbartolomeo@unime.it (G.B.); gbua@unime.it (G.B.); aferracane@unime.it (A.F.); tgervasi@unime.it (T.G.); rrando@unime.it (R.R.); dugog@unime.it (G.D.); spano@unime.it (N.S.); 2 Dipartimento di Scienze Chimiche, Biologiche, Farmaceutiche ed Ambientali (ChiBioFarAm), University of Messina, Viale Annunziata, 98168 Messina, Italy; linda89lv@gmail.com (R.L.V.); ssavoca@unime.it (S.S.); 3 Science4Life s.r.l., a Spin-Off of the University of Messina, 98168 Messina, Italy

**Keywords:** *Pelagia noctiluca*, Mediterranean jellyfish, chemical characterization, aquafeed and food supplements, sustainable fishing

## Abstract

The phylum Cnidaria is one of the most important contributors in providing abundance of bio- and chemodiversity. In this study, a comprehensive chemical investigation on the nutritional and nutraceutical properties of Mediterranean jellyfish *Pelagia noctiluca* was carried out. Also, compositional differences between male and female organisms, as well as between their main anatomical parts, namely bell and oral arms, were explored in an attempt to select the best potential sources of nutrients and/or nutraceuticals from jellyfish. With the exception of higher energy densities and total phenolic contents observed in females than males, no statistically significant differences related to the specimen’s sex were highlighted for the other compound classes. Rather, the distribution of the investigated chemical classes varied depending on the jellyfish’s body parts. In fact, crude proteins were more abundant in oral arms than bells; saturated fatty acids were more concentrated in bells than oral arms, whereas polyunsaturated fatty acids were distributed in the exact opposite way. On the other hand, major elements and trace elements demonstrated an opposite behavior, being the latter most accumulated in oral arms than bells. Additionally, important nutraceuticals, such as eicosapentaenoic and docosahexaenoic acids, and antioxidant minerals, were determined. Overall, obtained data suggest the potential employment of the Mediterranean *P. noctiluca* for the development of natural aquafeed and food supplements.

## 1. Introduction

The marine environment and its inhabitants are today recognized as an enormous reservoir of bioactive substances to be exploited for pharmaceutical and aquaculture applications, and as nutraceuticals as well [1,2,3,4]. Nonetheless, a number of anthropogenic activities have negatively affected marine ecosystems, inhibiting the derived services, and their precious bioactive resources as well [5,6,7,8]. 

In this scenario, Cnidaria such as scypho-, cubo-, and hydromedusae, simply referred to as ‘jellyfish’, have become issues of public concern, due to the huge proliferations taking place in coastal waters. The triggers of jelly ‘blooms’ are typically identified in (i) global warming [9,10], (ii) overfishing [11,12], and (iii) eutrophication [13,14].

When they bloom, jellyfish are infamous for having a negative impact on the structure and function of marine ecosystems and, consequently, on the related economic and social activities [10]. They grow fast and feed mainly on zooplankton and fish larvae, thus depressing many fish catches (e.g., anchovy and sardines) [15,16]. Also, intense Cnidaria outbreaks have reported to damage mariculture, as small specimens and their oral arms can enter net-pens, inducing gill hemorrhage and subsequent fish suffocation [17,18], and can clog the cooling water pipelines of coastal plants, with consequent power reductions and shutdowns [19]. Last but not least, due to the presence of nematocysts causing painful stings, jellyfish are notoriously venomous, and, in some cases, may compromise the general health status of the wounded swimmers and bathers, affecting negatively the tourism of many coastal localities [20].

Despite being traditionally considered as indicators of perturbed ecosystems and trophic dead ends, Cnidaria are nowadays object of a ‘paradigm shift’ reconsidering their ecological role. Indeed, in marine ecosystems, jellyfish (i) represent relevant prey and predators [21,22]; (ii) provide a shelter for certain juveniles fish species [23] and a habitat for various invertebrate organisms [24,25,26]; (iii) serve as hosts with photosynthetic algae, such as zooxanthellae, which, in turn, are critical to the ephyrae metamorphosis and the survival of jellyfish, supplying much of the carbon requirements [27,28,29]; and (iv) contribute to the nutritional cycling of the trophic web [30,31] 

Due to the high abundances and reproductive potential, paradoxically, jellyfish may be considered as value-added products, with various benefits for humans. Historically, jellyfish constitute a gourmet dish to be consumed in weddings and formal banquets, following a secret processing based on dehydration by water and alum. Umbrella are generally used for food consumption; however, oral arms from those species whose nematocyst toxin is relatively innocuous have been also considered [32,33]. According to recent estimates, at least 18 countries catch jellyfish for food, and a dozen or more countries are either exploring new fisheries or have been involved in jellyfish fisheries in the past [34]. Many countries do not report their catches of jellyfish explicitly to the Food and Agriculture Organization of the United Nations (FAO), as they include them either as ‘other aquatic animals’ (sea cucumbers, sea urchins, and edible jellyfish) or not at all. As a result, the average catch of ‘other aquatic animals’ reported by FAO in 2016, was approximately 938,500 tons (USD 6.8 billion) [35].

Species from the order Rhizostomeae, class Scyphozoa, are considered edible [36]. In this respect, *Rhopilema hyspidum* and *Rhopilema esculentum* represent relevant fishery and reared species in China [37], Malaysia [38], and Japan [39]; whereas *Stomolophus meleagris*, *Catostylus mosaicus*, and *Rhizostoma pulmo* are emerging fishery species respectively in the Gulf of Mexico [32], Australia [40], and Pakistan [41]. Overall, they are appreciated not only for the crunchy and crispy texture and taste, but also for their chemical composition, which ensures a low calorie intake, being low in carbohydrate, fat, and cholesterol. Although less desirable and not currently targeted at commercial scale, other Scyphozoa jellyfish (order Semaestomeae) such as Aurelia [42], Chrysaora [43], and Cyanea [44] have been consumed. Additionally, limited information suggests that cubozoans may be eaten in eastern regions, such as Taiwan [45]. Jellyfish have long been recognized also for their pharmaceutical value. *R. esculentum*, for example, is characterized by proteins from oral arms with a significant antioxidant activity [46]. Collagen from *S. meleagris* demonstrated to be an effective cure for rheumatoid arthritis in rats [47]; while collagen from *R. esculentum* showed antimelanogenic activity due to antioxidant properties and copper-chelating ability [48]. Jellyfish collagen might be also used in the biomedical area, for cartilage and bone reconstruction [49,50] and in the cosmetic field, for producing creams and lotions for skin care [51,52,53].

With this background, turning other yet unexplored jellyfish species from a nuisance into a sustainable resource becomes imperative for (i) controlling the size of jellyfish populations and (ii) maximizing the benefits related, but not limited, to their nutritional and nutraceutical potential.

*Pelagia noctiluca* (Scyphomedusae, Semaeostomae), also called “the mauve stinger”, is a pelagic jellyfish characterized by wide distribution, abundance, and relevant ecological role as well [54]. Massive outbreaks of *P. noctiluca* have increasingly occurred in the Mediterranean area largely as a result of anthropogenic activity, as evidenced by numerous studies from the late 1970s and 1980s, [55,56,57]. *P. noctiluca* has been consequently explored also for its toxicological relevance. Indeed, nematocyst morphology, toxicity [58,59], and activation [60], as well as symptoms and epidemiology of stings [61,62], have been addressed.

Nonetheless, the number of studies reporting data on the chemical composition of *P. noctiluca,* and its exploitation as nutraceutical source also appears to be quite low. To the best knowledge of the authors, only Milisenda and coworkers recently investigated different compositional aspects of *P. noctiluca* from the Strait of Messina for studying (i) sexual reproduction [63], (ii) trophic relationships [64], and (iii) dynamics of fish predation [65].

Therefore, in the present study, the nutritional value and nutraceutical value of *P. noctiluca* were investigated through the application of a variety of techniques. Specifically, biometric properties, gross energy, total polyphenol, and protein contents of the mauve stinger were determined. Also, fatty acid composition and major and trace element profile were screened. Compositional differences related to sex, and to the main anatomical parts, namely bell and oral arms, were explored in an attempt to select the best source(s) of nutrients and/or nutraceuticals from such species.

Scope of the work was not only to provide a chemical fingerprint of the mauve stinger, but also to encourage the fishing of the natural populations in the Mediterranean area for supporting its potential employment as feed supplementation and/or nutraceutical.

## 2. Results

### 2.1. Biometrics

**Figure 1:**
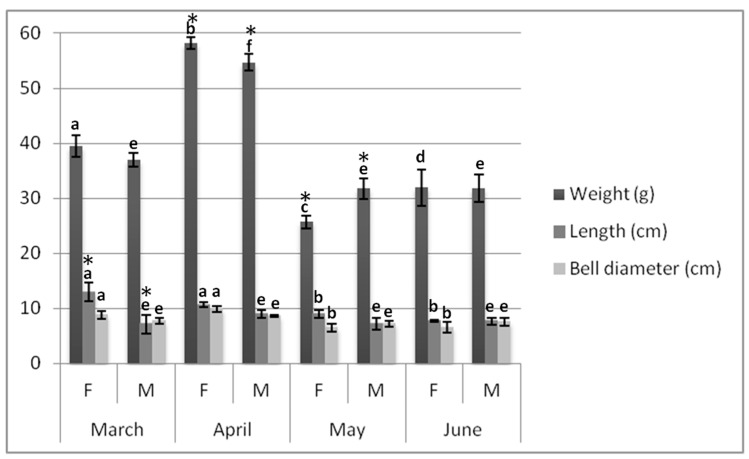
Fresh weight (g), bell diameter (cm), and length (cm) of jellyfish samples collected per sex and month. Data are reported as mean ± standard deviation (*n* = 50). According to the four sampling periods, female (or male) jellyfish marked by different letters for a given biometric characteristic, differ significantly (*p* < 0.05 by post hoc Tukey’s Honestly Significant Difference (HSD) test). According to sex specimens, female and male jellyfish marked by the asterisk for a specific parameter in the same sampling month differ significantly (*p* < 0.05 by post hoc Tukey’s HSD test). F = female. M = male.

Biometrics of *P. noctiluca*, caught from March to June, are reported in Figure 1; while in Table S1, the summary output of the two-way ANOVA analysis performed on investigated parameters, such as length (cm), bell diameter (cm), and weight (g) of the wet mass, is reported.

For each dependent morphometric variable, the two-way ANOVA analysis pointed out statistically significant interactions (*p* < 0.05) between the two independent variables (namely, specimen’s sex and sampling month) (Table S1).

Between March and April (2017), female organisms were characterized by length ranging between 10.6 and 13.0 cm (*p* > 0.05), and bell diameter between 8.8 and 9.9 cm (*p* > 0.05); while length and bell diameter of males were between 6.5 and 9.6 cm (*p* > 0.05) and 7.7 and8.6 cm (*p* > 0.05), respectively. Weight oscillated between 39.5 and 58.2 g for females (*p* < 0.05) and 37.0 and 54.7 g for male specimens (*p* < 0.05) (Figure 1).

Between May and June, minor lengths and bell diameters were recorded both for females (9.0–7.7 cm, *p* > 0.05, and 6.5–6.7 cm, *p* > 0.05, respectively) and males (7.1–7.7 cm, *p* > 0.05, and 7.2–7.5 cm, *p* > 0.05, respectively). Also, females, on average, had a lower weight distribution than males, with weights ranging from 25.7 to 31.9 g (*p* < 0.05) and from 31.7 to 31.8 g (*p* > 0.05), respectively (Figure 1).

Considering the different sampling periods, greater fluctuations of length, bell diameter and weight, were observed between March and April than June and May, both in female and male specimens. Additionally, from March–April to June–May, the investigated parameters were significantly reduced (*p* < 0.05) in female jellyfish. A significant reduction (*p* < 0.05) of wet mass was also observed in male jellyfish, but similar lengths (*p* > 0.05) and bell diameters (*p* > 0.05) were recorded throughout the sampling period.

Considering the sex specimens, in March, female and male medusae showed similar bell diameters (8.8 and 7.7 cm, *p* > 0.05) and weights (39.5 and 37.0 cm, *p* > 0.05) but significantly different lengths (13.0 and 7.2 cm, *p* < 0.05). In April and May, the trend changed as bell diameters of females and males were similar (9.9 and 8.6 cm, *p* > 0.05, and 6.5 and 7.2 cm, *p* > 0.05, respectively); while significantly different lengths (10.6 and 9.0 cm, *p* < 0.05, 9.0 and 7.1 cm, *p* < 0.05, respectively) and weights (58.2 and 54.7 g, *p* < 0.05, and 25.7 and 31.7 g, *p* < 0.05, respectively) were observed. Finally in June both female and male organisms showed similar lengths, bell diameters and masses (*p* > 0.05) (Figure 1).

### 2.2. Gross Energy Contents

**Figure 2:**
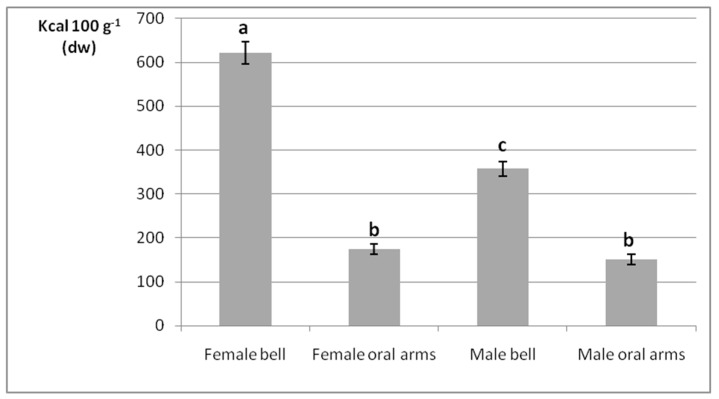
Gross energy densities of female and male jellyfish bells and oral arms. Data are reported as Kcal 100 g^−1^ on a dw basis, in terms of mean ± standard deviation (*n* = 3). Samples marked by different letters differ significantly (*p* < 0.05 by post hoc Tukey’s HSD test).

The gross energy densities of female and male jellyfish bells and oral arms obtained by bomb calorimetry are outlined in terms of Kcal 100 g^−1^ on a dry weight (dw) basis (Figure 2). The two-way ANOVA analysis revealed that the interaction between specimen’s sex and anatomical part (independent variables) was statistically significant (*p* < 0.05, Table S2). Overall, statistically significant differences were found independently of the considered body part (*p* < 0.05), as bell was characterized by higher energy content then oral arms, both in female and male medusae.

Additionally, considering the bell, statistically different energy values were detected between female and male specimens (*p* < 0.05). In fact, the sample from female bells showed the highest calorie value (621 Kcal 100 g^−1^), followed by the one from male bells (357 Kcal 100 g^−1^); whereas both male and female oral arms were characterized by inferior and nonsignificantly different calorie levels (174 and 151 Kcal 100 g^−1^, respectively, *p* > 0.05).

### 2.3. Crude Protein

**Figure 3:**
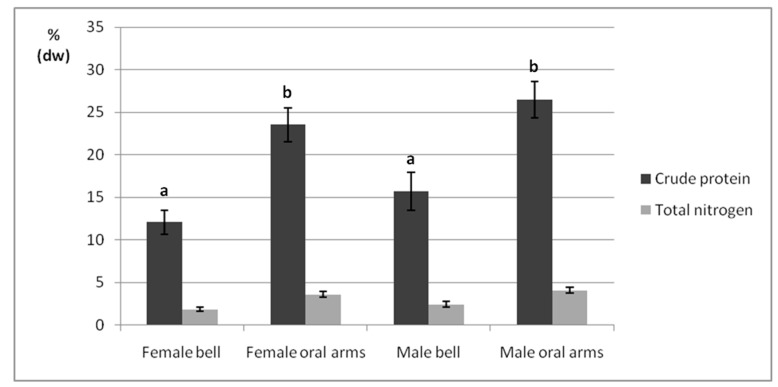
Total nitrogen and protein contents of female and male jellyfish bells and oral arms. Data are reported on a % dw basis, as mean ± standard deviation (*n* = 3). Samples marked by different letters differ significantly (*p* < 0.05 by post hoc Tukey’s HSD test).

Total nitrogen and protein contents of female and male jellyfish bells and oral arms are reported on a % dw basis by the bar graph in Figure 3. A nonstatistically significant interaction between the two independent variables (*p* > 0.05) was found (Table S3). Overall, investigated bells showed lower protein contents than oral arms (12.09–15.7% vs. 23.5–26.4%, *p* < 0.05), and nonstatistically significant differences were reported between female and male organisms (12.09–23.53% vs. 15.7–26.45%, *p* > 0.05).

### 2.4. Phenolic Compounds

**Figure 4:**
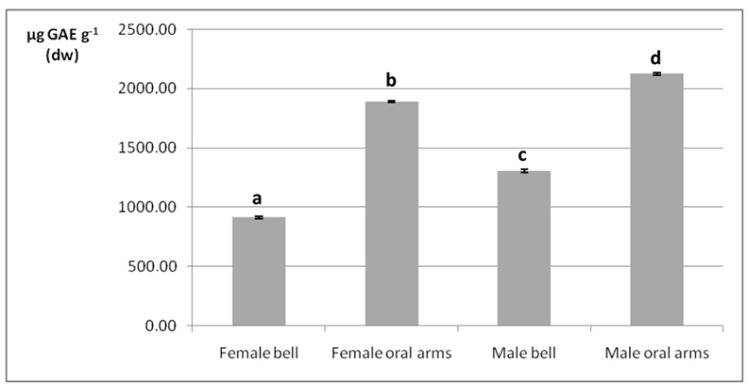
Total phenolic content (µg GAE g^−1^) determined in male and female jellyfishes’ bell and oral arms. Data are expressed as mean ± standard deviation (*n* = 3), on a dw basis. Samples marked by different letter differ significantly (*p* < 0.05 by post hoc Tukey’s HSD test).

As mentioned in the Materials and Methods section, at an initial stage of analysis, samples underwent two different preparation procedures, based on the use of PBS and methanol, respectively. Once the method was optimized, the first procedure was preferred, due to better analytical performance in terms of speed and efficiency. The UV spectrophotometric analysis determined the total phenolic content in dried bells and oral arms of male and female organisms in terms of µg of gallic acid equivalents (GAE) g^-^ on a dw basis (Figure 4). According to the two-way ANOVA test, the interaction of specimen’s sex and anatomical part was statistically significant (*p* < 0.05, Table S4). In general, phenolic compounds were more abundant in oral arms rather than in bells (1892–2126 µg GAE g^−1^ vs. 914–1303 µg GAE g^−1^, respectively, *p* < 0.05). Considering the same anatomical part (bell or arms), a statistically significant difference (*p* < 0.05) was also determined between the phenolic contents of male and female specimens: 1303.32 µg GAE g^−1^ vs. 914.22 µg GAE g^−1^; and 2126.56 µg GAE g^−1^ vs. 1892.69 µg GAE g^−1^, in males and females, respectively.

### 2.5. Fatty Acids

GC–MS analyses allowed us to determine around thirty fatty acid methyl esters (FAMEs), ranging from methyl hexanoate to methyl tetracosanoate, in dried samples from *P. noctiluca* (Table 1). With some exception, probably due to amounts below the detection limit, these FAMEs were common to each sample group, namely bells and oral arms of female and male specimens.

From a quantitative point of view, the main fatty acid classes varied depending on specimens’ body part rather than sex. In fact, saturated fatty acids (SFAs), representing 70% ca. in bells and 65% ca. in oral arms, respectively; monounsaturated fatty acids (MUFAs), accounting for 15% ca. in all samples (*p* > 0.05); and polyunsaturated fatty acids (PUFAs), constituting 14% ca. in bells and 19% ca. in oral arms (*p* < 0.05). Only slight and nonstatistically significant differences (*p* > 0.05) could be attributed to the specimen’s sex. For example, SFAs were slightly more abundant in male than female bells (70.6 vs. 69.5%, *p* > 0.05); MUFAs were slightly higher in female than male oral arms (15.8 vs. 14.6%, *p* > 0.05); and PUFAs content was slightly lower in male than female bells (13.8 vs. 15.4%, *p* > 0.05) and female than male oral arms (18.4 vs. 19.2%, *p* > 0.05).

It was not possible drawing conclusions on the distribution of single fatty acids according the organism’s anatomical part or sex. The only exception was represented by PUFAs of the ω6 and ω3 series, which showed to be constantly more abundant in oral arms than bells, regardless of the sex (Table 1). However, fatty acid fingerprints pointed out, as predominant components of the mauve stinger, lauric acid (3.16–4.69%), palmitic acid (32.87–36.46%), stearic acid (19.01–25.25%), arachidic acid (2.24–3.09%), lignoceric acid (1.11–1.95%), palmitoleic acid (0.61–2.38%), oleic acid (12.26–12.95%), linoleic acid (1.25–1.68%), arachidonic acid (4.23–6.67%), eicosapentaenoic acid (EPA, 4.96–6.40%), and docosahexaenoic acid (DHA, 2.36–4.02%). As can be seen in Table 1, the ω6/ω3 ratios determined in all samples were close to 1:1 (Table 1).

### 2.6. Major and Trace Element Profiles

The contents of four major elements (Na, Mg, K, and Ca), five essential trace elements (Fe, Cu, Zn, Mn, and Se), and five nonessential/potentially toxic trace elements (Cr, Ni, As, Cd, and Pb), were evaluated by Inductively Coupled Plasma-Mass Spectrometry (ICP-MS) in male and female jellyfish bells and oral arms, on a dw basis (Table 2).

Table S5 reports data of the validation procedure carried out by means of reference standards. The ICP-MS method showed good linearity for all the elements, with coefficients of correlation between 0.994 and 0.999. Acceptable recoveries between 93.49% (Ni) and 103.31% (Cr) were obtained. Evaluated in terms of RSD%, precision (intraday repeatability) and intermediate precision (interday repeatability), resulted to be within the range of 2.02 to 6.53%, and below 8.52%, respectively.

Major element signatures varied most in dependence of the organ rather than sex (Table 2). Overall, these metals appeared to bioaccumulate mainly in bells than the respective oral arms (*p* < 0.05) by the decreasing order of Na > Mg > Ca ≈ K, regardless of female and male sex (*p* > 0.05). As a result, Na was characterized by the highest levels (6544–8079 mg 100 g^−1^ for bells; 3877–3740 mg 100 g^−1^ for oral arms); while K was the less abundant one, showing contents inferior by one order of magnitude (196–229 mg 100 g^−1^ for bells; 126–143 mg 100 g^−1^ for oral arms) (Table 2).

Dealing with essential trace elements, they were found in the decreasing order of Fe > Cu > Zn > Mn > Se, in both male and female bells and oral arms. Fe was characterized by a behavior similar to major elements, since it was more abundant in bells (1309–1465 µg 100 g^−1^) than oral arms (854–1085 µg 100 g^−1^) (*p* < 0.05), regardless of specimens’ sex. However, Cu, Zn, Mn, and Se completely inverted such trend, showing to be more abundant in oral arms than bells (*p* < 0.05). Also, Zn and Mn contents showed a slight dependence on the specimens’ sex, though in a nonstatistically significant manner (*p* > 0.05), since males showed to most bioaccumulate such elements than females in both bell and oral arms. Being not yet determined in any jellyfish species, Se levels ranged from 31.2 to 46.2 µg 100 g^−1^ in bells and from 100 to 115 µg 100 g^−1^ in oral arms (Table 2).

Nonessential/toxic trace elements were reported in the decreasing order of Cr ≈As > Ni > Pb > Cd. Such elements were slightly higher in oral arms than in bells (*p* > 0.05 in almost all cases), and, with the exception of Pb, marginally more bioaccumulated in male organisms than female ones (*p* > 0.05) as well. Cr and As varied respectively between 401 and 573 µg 100 g^−1^ and 412 and 528 µg 100 g^−1^ in female and male bells; while female and male oral arms reported Cr and As levels of 668 to 631 µg∙100 g^−1^ and 690 to 663 µg∙100 g^−1^, respectively. Contrary to Cr and As, Pb exhibited the lowest contents in both bells (140–117 µg 100 g^−1^) and oral arms (154–132 µg 100 g^−1^), with comparable values between female and male specimens (Table 2).

## 3. Discussion

Despite of the short period (four months) of sample collection, morphometric parameters, namely body length, bell diameter, and wet mass were measured in order to integrate the knowledge about *P. noctiluca* in the Mediterranean Sea, particularly in the peculiar environment of the Strait of Messina.

As already reported by Sandrini et al. [66], the metabolism of *P. noctiluca* is directly proportional to the sea temperature, so that a temperature increase results in an increased metabolism rate and food requirement and accelerates growth. Also, enhanced food availability, in terms of zooplankton and ichthyoplankton blooms, typically occurring in the Strait of Messina during spring [67,68,69], may be responsible of the higher weights recorded in female and male organisms, in agreement with the findings reported by Rosa et al. for *P. noctiluca* caught in the same seawaters [70]. Clearly, during March and April female organisms had the highest weight as they reached full gonads’ maturity, resulting to be also sparingly bigger than males. In fact, biometrical and reproduction analyses performed by Rosa and coworkers [70] suggest that in the Strait of Messina, *P. noctiluca* reproduces with a maximum peak in late autumn/early winter. In this season, medusae diameter rapidly increases and most of the females are mature. They continue to grow and spawn until March–April, and in May they can reach the end of their life cycle, bringing empty, small, and slightly colored gonads. This could explain our observations on the decrease of weight during May and June in parallel to bell diameter reduction, especially in the female organisms.

As concerns bell diameter, findings of the present study are also in accordance with those reported by Rosa et al. [70]; in that case, bell diameter and water temperature were negatively correlated, providing an explanation for bell’s reduction as the weather approaches the warm climate (May–June). Additionally, the mean bell’s reduction recorded in May and June may be related to the presence of different age jellyfish groups, including younger (and consequently smaller) individuals, as suggested by Milisenda and colleagues [63]. Even small variations of bell size had a remarkable effect on body weight, as previously reported by Lilley et al. [71], where bell diameter accounted for 98% of the variation in wet mass.

As a basic nutritional aspect, the gross energy content of *P. noctiluca* was first evaluated. The greater calorie values of bells compared to oral arms, both in female and male specimens, was presumably due to the presence of the high-calorie gonads. In particular, energy density of female bell exceeded the male counterpart, due to higher carbohydrate, lipid, and protein contents of eggs next to reproduction maxima period [63]. The energetic value of jellyfish was barely addressed with respect to other common planktonic preys. Milisenda and coworkers carried out the energy count of *P. noctiluca*’s gonads and oral arms, the latter resulting in similar values in male and female specimens (~52.3 Kcal 100 g^−1^) [65]. Doyle et al. [72] assessed the gross energy densities among several jellyfish species from Ireland (*Cyanea capillata*, *Rhizostoma octopus* and *Chrysaora hysoscella*) and between different tissues (bell and oral arms) within the same species as well. Besides the fact that each species was characterized by its own energy value, it was pointed out that oral arms were coherently characterized by higher gross energy contents than bells in any case. Indeed, gonads were not considered in these calorie counts, being excised from the respective bells, and separately assessed. Recently, higher gross energy densities in edible Malaysian jellyfish, such as *Rhopilema esculentum, Acromitus hardenbergi*, and *Rhopilema hispidum*, have been reported, with calorie values within in the range of 211 to 97 kcal∙100 g^−1^ for bells and 282 to 200 kcal∙100 g^−1^ for oral arms [38]. However, as contemplated by the experimental plan, gonad-provided bells were apparently investigated in such study.

Proteins are known to constitute the largest portion of the organic matter present in jellyfish dried samples, which typically contain only lower amounts of either lipids or carbohydrates [70,71,73]. Being presumably attributable to the dominant structural collagen distributed throughout the jellyfish body, the peculiar protein fraction is an appealing source of essential nutrients, thus, making such marine invertebrates potential suitable for food/feed purposes [46,74,75]. Looking at *P. noctiluca* samples, oral arms reported higher crude protein content than bells, regardless of specimen’s sex, probably not only because of the abundant structural collagen, but also due to the labile protein toxins present in the numerous nematocysts typically present in oral arms. Significant variations between the different body parts were confirmed also in *C. capillata*, *R. octopus*, and *C. hysoscella*, with oral arms contents (13.1–34.1%) higher than the ones detected in gonad excised bells (5.2–11.2%) [72]; and in *R. esculentum*, *A. hardenbergi*, and *R. hispidum* species, where bell and oral arms proteins were within the range of 19.95 and 38.12% and 33.6 and 53.8%, respectively [38]. However, the qualitative and quantitative distribution of proteins both in bells and oral arms of the Mediterranean *P. noctiluca* should be further explored by appropriate proteomic techniques.

Polyphenols are widely acknowledged antioxidant compounds, widespread in nature and particularly concentrated in plants. When referring to polyphenols from the marine environment, literature indicates micro- and macroalgae as the main sources for such compounds [76,77,78]. Nevertheless, in consideration of the scope of the present study, namely to investigate the nutritional and nutraceutical properties of the mauve stinger, the determination of the total polyphenol content was reasonably carried out. Previous reports on total polyphenols in jellyfish species are very few. Leone et al. [74,75] reported the total phenolic contents of three jellyfish species, none belonging to *Pelagia* genus. In two of them, ~1800–2000 µg GAE g^−1^ (dw) were determined. Such findings were quite in accord with the present report. The total phenolic content determined in *P. noctiluca* is only a preliminary and rough screening that should be followed by a targeted qualitative analysis for the identification of the specific polyphenolic structures. In fact, it has been speculated that amino acidic residues from the protein fraction could contribute to the value of the total phenolic content [74].

The results obtained from the analyses of fatty acids can be hardly compared with previous studies, since *P. noctiluca* results to be underinvestigated with respect to this issue. Mastronicolis et al. reported 0.19% *ww* of total lipids, 41.7% of which were free fatty acids [79]; whereas Cardona et al. analyzed the fresh mass of *P. noctiluca* obtaining a fatty acid composition roughly represented by 70% palmitic acid, 15% pentadecanoic acid, 6% EPA, and 2% DHA [80]. Approximately, the fatty acid profiles here presented are in accordance with those reported for other jellyfish species, collected in the Mediterranean Sea and investigated with the same analytical procedure here applied [74,80,81].

Notoriously, essential fatty acids belonging to the ω-6 and ω-3 groups, and are significantly present in a variety of vertebrate and invertebrate marine species, and, when correctly balanced, confer a relevant nutraceutical value to the source, which they come from [82]. As can be observed in Table 2, essential ω-3 fatty acids, such as EPA and DHA, and essential ω-6 fatty acids, such as linoleic acid, were determined also in the Mediterranean *P. noctiluca*, being more abundant in oral arms than bells. Since pioneer works on the positive correlation between essential fatty acids and human health, different schools of thought have arisen during the years, attributing a higher healthy power one time to ω6 acids, another to ω3 acids. However, in the last decades, scientists concluded that it is the balance between ω6 and ω3 that promotes normal development and preserves homeostasis. Ideally, this ratio should be 1:1, due to the observation of chronic diseases especially in those individuals having a diet deficient of ω3- fatty acids and, consequently, with an unbalanced ω6/ω3 ratio [83]. In this respect, all samples of *P. noctiluca* reported a correct balance of the two classes of fatty acids, thus confirming its nutraceutical value. In particular, bell and oral arms from female organisms showed the ratios closest to 1:1 (respectively, 0.89 and 0.80).

The screening of major and trace elements provided further insights on the nutritional and nutraceutical value of *P. noctiluca*. Jellyfish bioaccumulates and transfers essential minerals and trace elements from lower trophic levels to high-order fish predators, having a key role in balancing any potential nutritional shortfall of the food chain. The same applies to nonessential and potentially toxic elements, which could instead represent a threat for the health of marine ecosystems, and, not least, human consumers [84,85]. To date, few published data dealt with inorganic elements in this gelatinous zooplankton, mainly to test the environmental quality of coastal systems [81,84,85,86,87], rather than for food/feed purposes [38].

According to obtained data, jellyfish bell exhibited higher Na, Mg, K, and Ca levels than oral arms, probably because of the buffering activities carried out to maintain the osmotic balance and, thus, the floating capacity of the bell [38]. Although such a mineral distribution was confirmed also by previous works on different jellyfish species from Portuguese (*Catostylus tagi)* and Australian (*Cassiopea* sp.) coasts [81,84], not comparable contents were detected, because of clear taxonomic and ecological reasons. For example, Mg in bell and oral arms belonging to *C. stagi* and *Cassiopea* sp. was equal to 328 mg 100 g^−1^ and 240 mg 100 g^−1^ [81], and 74.4–129.7 mg 100 g^−1^ and 73.4–125.7 mg 100 g^−1^, respectively [86]. Ca levels distributed between bells and oral arms within the ranges 25.8 to 46.6 mg 100 g^−1^ and 27.2 to 44.6 mg 100 g^−1^ [84] in the Australian *Cassiopea* sp.; whereas, in the Portoguese *C. stagi*, bell and oral arms showed Ca contents equal to 1026 mg 100 g^−1^ and 736 mg 100 g^−1^ [81].

Trace elements, such as Fe, Zn, Cu Mn, and Se, are well known to be both limiting nutrients and toxicants. In fact, they are essential for the metabolism of vertebrate and invertebrate marine organisms, as they constitute a variety of metalloproteins and antioxidant enzymes and play a key role in cellular detoxification activity, but, at the same time, they become toxic at high concentrations, leading to damaging oxidative processes [88]. In the same way as for major elements, the comparison of trace element levels from *P. noctiluca* with those from other jellyfish species addressed in previous works [81,84,85] becomes challenging. In fact, an increasing discrepancy seems to appear dealing thoroughly with getting smaller contents of essential trace elements; however, similarly to *P. noctiluca*, also other species, such as *C. stagi* [81], *Cassiopea* sp. [84], and *Cotylorhiza tuberculata* [85], showed to more bioaccumulate such trace elements in oral arms than bells. These distribution patterns could be related to the metal uptake via food, occurring by oral arms directly involved in suctorial feeding, and fine filtering functions [89]. Assuming that the elemental requirements of an organism, including the human being, are driven almost entirely by utility (i.e., cellular function, with shifts in biological requirements decoupled from corresponding environmental abundances), and that they should be assessed in depth case-by-case, it is reasonable to hypothesize that both essential major and trace elements found in the Mediterranean *P. noctiluca*, may be exploited for developing natural food and aquafeed supplements. In the latter case, other jellyfish species, such as *A. aurita* and *Chrysaora pacifica*, have already been demonstrated to support the growth and survival of specific farmed species, thanks also to a peculiar metal profile [90].

The main source of nonessential and potentially toxic elements, such as Cr, As, Ni, Pb, and Cd, typically comes from anthropogenic activities, negatively impacting the health status of marine environments. Previous works have already pointed out that different jellyfish species can bioconcentrate these harmful elements and reflect a time-integrated measured of their levels in the water, demonstrating to be useful bioindicators of coastal environments [84,85]. The uptake and accumulation of nonessential/toxic elements in *P. noctiluca* varied, although in nonsignificant manner, between selected tissues and sex specimens. However, as the present study does not rely on an ecotoxicological basis, and no literature on the pollutant accumulation capacity of the Mediterranean *P. noctiluca* has been yet produced, no conclusion will be drawn about the health status of such coastal areas. Nonetheless, obtained data could be useful for stressing on the bioaccumulating capacity of jellyfish, other than constituting an input for future environmental studies on such species.

Concerning the toxicological value of *P. noctiluca* as aquafeed and food supplement, it is useful to mention the Commission Regulation (EU) N. 744/2012 [91] setting the limits of heavy metals in animal feeds, and the Commission Regulation (EC) N. 629/2008 [92], amending the Regulation (EC) N. 1881/2006 and fixing the maximum levels of heavy metals in food supplements. Indeed, according the EU Regulation N. 744/2012, the investigated samples were characterized by means concentrations of As and Pb (5.58 and 1.35 mg kg^−1^) well within the maximum contents set at 10 and 5 mg kg^−1^ for a complete feed, respectively, and at 10 and 10 mg kg^−1^ for a complementary feed, respectively, in every case with a moisture content of 12%. On the other hand, according the EC Regulation (EC) N. 629/2008, *P. noctiluca* showed mean levels of Pb and Cd (1.35 and 0.45 mg kg^−1^), well below the limits set respectively at 3.0 and 1.0 mg kg^−1^.

As a result, potential aquafeed and food supplements based on *P. noctiluca* from the Strait of Messina would be safe for farmed fish and human consumers, in terms of toxic heavy metals.

The pioneering chemical composition data of *P. noctiluca* herein discussed may be a valuable starting point for acknowledging its nutritional and nutraceutical properties for food and aquafeed purposes.

Concerning food applications, it has been widely discussed the relevant value of certain jellyfish species as food for human consumption in Asian countries. Additionally, a specific protein fraction of the edible *R. esculentum* with high antioxidant power [93] and a new bioactive polysaccharide isolated from edible jellyfish [94] were proposed for developing novel and natural food supplements.

Regarding aquafeed field, Marques et al. recently revealed that all developmental stages of *Aurelia* sp. were accepted as a feed source by *Sparus aurata* [73]; *Aurelia aurita* was also demonstrated to be a valid feed source for *Thamnaconus modestus*, when other common preys were not visible [95], and for rearing commercial fish such as *Pampus argenteus* [96]. Furthermore, Milisenda and colleagues proposed *P. noctiluca* as valuable food source for fish predators, such as Boops boops, in the Strait of Messina, due to an increased energy content during the period of gonad maturation, and to the high available biomass present during blooms [65].

Clearly, before getting into any practical application of *P. noctiluca*, other in vitro studies will be necessary for firstly developing the most suitable procedures of biomass processing and venom neutralization. Then, if the applications turned out to be economically and realistically feasible, further research will be required to investigate (i) the in vivo toxicity and effectiveness of food/aquafeed supplements and (ii) the potential food web cascading effects due to the harvesting of wild populations.

## 4. Materials and Methods

### 4.1. Chemicals

All materials and reagents employed for the histological analyses were supplied by Bio-Optica Milano S.p.a. (Milan, Italy). The following reagents were all provided by Sigma-Aldrich (Milan, Italy): sulfuric acid (95–98% purity), sodium hydroxide, boric acid, hydrogen chloride, benzoic acid, and phosphate-buffered saline (PBS). The Kjeldahl catalyst was supplied by Carlo Erba (Milan, Italy). n-Heptane was purchased from PanReac AppliChem (Barcelona, Spain). Potassium hydroxide, chloroform, methanol, sodium carbonate anhydrous, Folin Ciocalteau’s reagent, stock standard solution of Re (1000 mg L^−1^ in 2% HNO_3_), gallic acid, and stock standard solutions of Sc, Ge, In, and Bi (1000 mg L^−1^ in 2% HNO_3_) were from Fluka (St. Gallen, Switzerland). Nitric acid (65%, *v*/*v*) was of Suprapur grade (Mallinckrodt Baker, Milan, Italy). Ultrapure water (<5 mg L^−1^ TOC) was obtained from a Barnstead Smart2Pure 12 water purification system (Thermo Scientific, Milan, Italy).

### 4.2. Sample Collection

Specimens of *P. noctiluca* were sampled from the Strait of Messina, South Italy. In this peculiar environment, the mauve stinger can be found in the Strait of Messina during the whole year, reaching the highest diffusion in the period of March to June. Approximately 400 adult specimens were collected from March to June 2017 (~100 organisms per month), in the coastal waters of Capo Peloro (Messina). Adult jellyfish samples were taken by means of a small net from water’s edge, put in tanks filled with seawater, and immediately transported to laboratory for morphometric measurements and sex and chemical determinations. The experimental protocol was developed in accord with the ethical standards reported in the European Directive 2010/63/EU on the protection of animals used for scientific purposes [97].

### 4.3. Biometrics and Sex Determination

**Figure 5:**
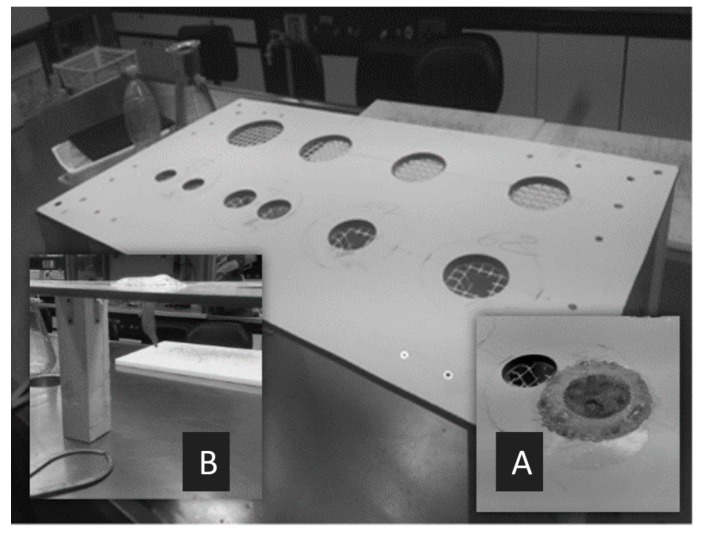
Lab-made apparatus for morphometric measurements. (**A**) Jellyfish were placed inside the most appropriate circular hole, chosen among different predefined measures. (**B**) Length was measured from the top of the bell to the end of the longest oral arm.

Maximum length, bell diameter, and total weight (fresh mass) of each jellyfish sample were measured with the support of a ruler and of a lab-made apparatus, shown in Figure 5. Each specimen was placed inside the most appropriate circular hole, chosen on the basis of bell diameter (Figure 5A). Oral arms were left suspended on the other side of the flat surface. Length was measured from the top of the bell to the end of the longest oral arm, along the oral–aboral axis (Figure 5B). Bell diameter was considered as the maximum distance between distal tips of opposed interradial rhopalia. Biomass (*w*/*w*) was measured after washing medusae with distilled water for salt removal. For each specimen, the sex was determined by inspection of the gonads through a stereomicroscope (Carl Zeiss Stemi SV11): male individuals presented purple follicles, while female subjects were characteristically pink-red colored, and crowded by Milisenda et al. [65]. When identification of sex resulted to be challenging or uncertain, histological assessments were carried out following a standard protocol [98].

### 4.4. Sample Lyophilization

After biometrical measurements and sex determinations were carried out, the organisms were sacrificed separating the bells by the manubrium bearing the oral arms, with the help of a Teflon knife, avoiding metal cross-contamination. Bells and oral arms were grouped to provide four different pools (*n* = 200 each), as reported in Table 3. Each pool was weighed, cleansed thoroughly with distilled water, minced, and lyophilized by an Alpha 1-2/LD Plus freeze dryer (Martin Christ, Osterode, Germany), for 72 h at −55 °C using a chamber pressure of 0.110 mbar. Then, the freeze dried pools were weighed, and stored at −20 °C until use. For each pool, a moisture content of ~95% *w*/*w* and a yield of 5% *w*/*w* were assessed.

### 4.5. Gross Energy Assessments

To measure the gross energy content, a benchtop isoperibol calorimeter (Parr^®^ 6200 Oxygen Bomb Calorimeter, Parr Instrument Company, Moline, IL, USA) was employed.

Approximately 1 g of each powdered pool was placed in a 1108 model oxygen bomb. To determine the gross energy densities of bombed samples, the calorimeter chamber was previously calibrated for the heat of combustion of 1 g of benzoic acid (26.46 kJ g^−1^), under controlled and reproducible operating conditions. In fact, the known amount of heat produced by the combustion of the calibration standard determined the energy equivalent (W) per change in water temperature between initial and postcombustion of the sample (ΔT). Therefore, the energy content of each sample (ES) was calculated as follows

ES = W × ΔT/exact sample weight

Each sample pool was run in triplicate. Results were expressed as Kcal 100 g^−1^, dw.

### 4.6. Crude Protein

The crude protein content was determined using the AOAC Official Method 976.05 (automated Kjeldahl method) [99]. Approximately 1 g of each powdered pool was separately digested by the SpeedDigester K-439 (Büchi, Switzerland) and then analyzed by the KjelMaster System K- 375 (Büchi, Switzerland) and equipped with a scrubber of gases and vapors (Scrubber K-415, Büchi, Switzerland). For the calculation of the % protein in a sample, the obtained % nitrogen was multiplied by a conversion factor of 6.5. Each determination was conducted in triplicate.

### 4.7. Total Phenolic Content

As a preliminary part of method development, an initial group of samples was subjected to two different extraction procedures: (i) with PBS (phosphate-buffered saline), pH 3.5 and (ii) with 80% methanol [74]. Aliquots (1.0 g) of each lyophilized sample pool were added with 16 mL of PBS and shaken for 2 h at 4 °C, in one case; with 16 mL of methanol and shaken for 16 h at 4 °C, in the other case. Successively, samples were homogenized at 9000 rpm and 4 °C for 30 min. To 1.0 mL of supernatant, 5.0 mL of Folin–Ciocalteau reagent and 5.0 mL of sodium carbonate (20%) were added. The resulting solution was kept in the dark for 2 h, and later analyzed at the UV–Vis spectrophotometer, model UV-2401PC (Shimadzu, Milan, Italy). The wavelength of absorbance was set at 760 nm. A 5-point calibration plot was built up by using solutions of gallic acid in methanol in the range 50 to 2000 µg mL^−1^. Each point corresponded to three replicates.

### 4.8. Fatty Acids

The extraction of fatty acids was carried out following the procedure reported by Bligh & Dyer [100]. An aliquot (10.0 mg) of each lyophilized pool was homogenized with 1.0 mL of chloroform (CHCl_3_) and 3.0 mL of methanol (MeOH). This mixture was then added with 1.0 mL of MeOH and 1.0 mL of water, homogenized, let to settle, and filtered through paper. After settling, the filtrate was centrifuged at 3000 rpm for 15 min. Two layers were formed: the bottom one (CHCl_3_), containing the isolated lipids, was transferred to a rotating evaporator, model P/N Hei-VAP Precision ML/G3 (Heidolph Instruments GmbH & Co., Schwabach, Germany). The upper layer (MeOH/H_2_O) was subjected again to all the steps above described in order to reach an exhaustive extraction. For the fatty acid methylation, the dried lipidic extract was recovered through addition of 1 mL hexane, then added with reagent (CH_3_OH/H_2_SO_4_, 9:1), and heated at 100 °C for 1 h. The hydrocarbon layer was collected and injected into the GC instrumentation. Qualitative analyses were carried out in a GCMS-TQ8030 (Shimadzu, Kyoto, Japan) system equipped with an AOC-20i autosampler and a capillary column Supelco SLB-IL100 (60 m × 0.25 mm, film thickness 0.20 µm). GC conditions were set as follows: injector, 280 °C; injection volume: 1.0 mL; head pressure: 26.7 kPa; carrier gas: He, at a linear velocity of 30.0 cm/s (constant); split ratio 1:100; oven temperature program: 50–280 °C at 3 °C/min, held 10 min. MS conditions: operation mode was in full scan; ion source and interface temperatures were 220 °C and 250 °C, respectively; and scan mass range was 40 to 400 *m*/*z*. For FAMEs identification, a triple means methodology was used: (i) spectral matching with Wiley and NIST databases; (ii) co-injection with standards (Supelco 37 component FAME mix, Supelco, St. Louis, MO, USA); and (iii) comparison with literature data [101]. Data handling was performed by GCMS solution software. Quantitative analyses were carried out on a Master GC-DANI system, equipped with a capillary column Supelco SLB-IL100 (60 m × 0.25 mm, film thickness 0.20 µm). Oven temperature program: 120–200 °C at 1 °C min^−1^ (10 min). Injector and FID temperatures were respectively set at 220 and 240 °C. Carrier gas was He, at a constant linear velocity of 30.0 cm s^−1^. FID conditions: sampling frequency: 25 Hz; gases: makeup (He), 25 mL min^−1^; H_2_, 40 mL min^−1^; air, 280 mL min^−1^. Data were processed through the Clarity software (Dani). All determinations were run in triplicate.

### 4.9. Elemental Analysis

Approximately 500 mg of each lyophilized pool were digested with 10 mL of HNO_3_, exploiting a closed-vessel microwave digestion system Ethos 1 (Milestone, Bergamo, Italy) equipped with PTFE vessels. The mineralization was carried out by setting the following temperature program; 0–200 °C in 10 min (step 1), 200 °C held for 5 min (step 2), and 200–220 °C in 5 min (step 3), with a constant microwave power of 1000 W. Due to the expected very high salt concentrations, each digested pool was diluted by ultrapure water with a dilution factor of 1000, and stored at 4 °C until ICP-MS analysis. A quadrupole ICP-MS iCAP Q (ThermoScientific, Waltham, MA, USA), equipped with an ASX-520 autosampler (Cetac Technologies Inc., Omaha, NE, USA), was employed for the analytical determinations. The ICP-MS operating conditions are shown in Table 4. All samples were analyzed in triplicate, along with blanks to check for any loss or cross contamination. For quantitative purposes, the external calibration procedure was carried out with the help of multielemental standard solutions. For building up six point calibration plots, six different concentrations were evaluated per analyte, and each point resulted from triplicate extractions and analyses [102]. For method validation, a linear least-square regression analysis of the calibration graphs was performed to check for the linearity between the instrumental response and the nominal concentration of each elemental standard. The intra-assay and interassay variabilities were determined by quantifying three replicates on the same day and six consecutive days, respectively.

### 4.10. Statistical Analysis

With the exception of biometrics, all data are reported as mean ± standard deviation of triplicate measurements. Statistical analysis was conducted by the SPSS Statistics Software v. 21.0 (SPSS Inc., Chicago, IL, USA), performing for each investigated variable a two-way analysis of variance (ANOVA) followed by the Tukey’ s honestly significant difference (HSD) post-hoc test. Level of significance was set at *p* < 0.05

## 5. Conclusions

In this study, male and female specimens of *Pelagia noctiluca* from the Strait of Messina were chemically characterized with respect to their nutritional and nutraceutical properties, taking into consideration both their bells and oral arms. Overall, the gross energy content was higher in bells rather than oral arms, while proteins and total phenolics were inversely concentrated. The fatty acid composition included important MUFAs, such as oleic acid, and PUFAs, such as EPA and DHA. Also, antioxidant inorganic elements were revealed. Both chemical classes varied in dependence of the investigated jellyfish part. In fact, SFAs were more concentrated in bells than oral arms, whereas PUFAs were distributed in the exact opposite way. MUFAs appeared to be equally present in both organs. On the other hand, major elements and trace elements demonstrated an opposite behavior, being the latter most accumulated in oral arms than bells.

With the exception of the significantly high energy contents and total phenolic contents observed in female organisms, no remarkable differences that could be ascribed to the variable “sex” were highlighted for the other compound classes. With the appropriate precautions, obtained results may support the potential employment of *P. noctiluca* as aquafeed or food supplement.

## Figures and Tables

**Table 1:** Fatty acid methyl esters determined in the bells and oral arms of female and male specimens from *Pelagia noctiluca*. Data are reported on a dw basis, as average Gas Chromatography-flame ionization detector ( GC-FID) peak area percent ± standard deviation (*n* = 3).

Fatty Acid	Female Bell (%)	Female Oral Arms (%)	Male Bell (%)	Male Oral Arms (%)
C6:0	-	0.06 ± 0.01 ^a^	-	0.04 ± 0.01 ^b^
C8:0	0.98 ± 0.07 ^a^	0.65 ± 0.03 ^b^	0.79 ± 0.03 ^c^	0.61 ± 0.03 ^b,d^
C10:0	1.27 ± 0.16 ^a^	0.85 ± 0.03 ^b^	0.65 ± 0.05 ^c^	0.88 ± 0.03 ^b,d^
C12:0	4.69 ± 0.17 ^a^	3.87 ± 0.09 ^b^	3.16 ± 0.17 ^c^	4.47 ± 0.09 ^a,d^
C13:0	0.18 ± 0.02 ^a^	0.21 ± 0.01 ^b^	0.18 ± 0.02 ^a^	0.15 ± 0.01 ^a^
C14:0	0.98 ± 0.14 ^a^	0.80 ± 0.06 ^b^	0.94 ± 0.07 ^a,c^	0.86 ± 0.03 ^b,d^
C15:0	0.81 ± 0.03 ^a^	0.63 ± 0.03 ^b^	0.79 ± 0.04 ^c^	0.69 ± 0.05 ^b,d^
C16:0	36.46 ± 0.1 ^a^	33.84 ± 0.19 ^b^	32.87 ± 0.50 ^c^	34.32 ± 0.18 ^b,d^
C17:0	0.40 ± 0.04 ^a^	0.65 ± 0.04 ^b^	0.53 ± 0.06 ^c^	0.94 ± 0.03 ^d^
C18:0	19.60 ± 0.37 ^a^	20.21 ± 0.21 ^a^	25.25 ± 0.25 ^b^	19.01 ± 0.06 ^c^
C20:0	2.51 ± 0.07 ^a^	2.42 ± 0.07 ^a^	3.09 ± 0.06 ^b^	2.24 ± 0.07 ^c^
C22:0	0.31 ± 0.06 ^a^	0.20 ± 0.02 ^b^	0.21 ± 0.02 ^b^	0.24 ± 0.01 ^b^
C23:0	0.06 ± 0.01 ^a^	0.15 ± 0.03 ^b^	0.22 ± 0.03 ^c^	0.18 ± 0.02 ^b^
C24:0	1.27 ± 0.09 ^a^	1.23 ± 0.04 ^a^	1.95 ± 0.08 ^b^	1.11 ± 0.03 ^c^
***SFAs***	***69.53 ± 0.77*** ^a^	***65.76 ± 0.91*** ^b^	***70.64 ± 1.19*** ^a,c^	***65.76 ± 0.15*** ^b,d^
C14:1	0.70 ± 0.03 ^a^	0.47 ± 0.07 ^b^	0.68 ± 0.06 ^a,c^	0.40 ± 0.02 ^b,d^
C15:1	0.18 ± 0.04 ^a^	0.16 ± 0.01 ^a^	0.15 ± 0.01 ^a^	0.09 ± 0.01 ^b^
C16:1	1.32 ± 0.09 ^a^	1.87 ± 0.09 ^b^	2.38 ± 0.10 ^c^	0.61 ± 0.03 ^d^
C17:1	0.21 ± 0.03 ^a^	0.18 ± 0.02 ^a,b^	0.15 ± 0.02 ^b,c^	0.09 ± 0.01 ^d^
C18:1 n-9	12.26 ± 0.11 ^a^	12.71 ± 0.22 ^a^	12.48 ± 0.34 ^a^	12.95 ± 0.10 ^a^
C20:1 n-9	0.10 ± 0.01 ^a^	0.06 ± 0.01 ^b^	0.06 ± 0.01 ^b^	0.10 ± 0.02 ^a^
C22:1 n-9	-	0.06 ± 0.01 ^a^	-	0.08 ± 0.01 ^a^
C24:1 n-9	0.45 ± 0.04 ^a^	0.32 ± 0.03 ^b^	0.30 ± 0.03 ^b^	0.32 ± 0.04 ^b^
***MUFAs***	***15.22 ± 0.32*** ^a^	***15.83 ± 0.42*** ^a^	***16.19 ± 0.53*** ^a^	***14.64 ± 0.07*** ^b^
C18:2 n-6	1.42 ± 0.09 ^a^	1.68 ± 0.04 ^a^	1.25 ± 0.16 ^b^	1.48 ± 0.05 ^a^
C18:3 n-6	0.09 ± 0.02 ^a^	0.11 ± 0.04 ^a^	0.08 ± 0.01 ^a^	0.10 ± 0.02 ^a^
C18:3 n-3	0.24 ± 0.03 ^a^	0.31 ± 0.05 ^b^	0.13 ± 0.04 ^c^	0.24 ± 0.02 ^a,d^
C20:2 n-6	0.09 ± 0.01 ^a^	0.17 ± 0.02 ^b^	0.07 ± 0.03 ^c^	0.18 ± 0.01 ^b,d^
C20:3 n-6	-	0.04 ± 0.02 ^a^	-	0.05 ± 0.01 ^a^
C20:4 n-6	5.45 ± 0.22 ^a^	6.61 ± 0.23 ^b^	4.23 ± 0.31 ^c^	6.67 ± 0.24 ^b,d^
C20:5 n-3	5.79 ± 0.15 ^a^	5.82 ± 0.13 ^a^	4.96 ± 0.09 ^b^	6.40 ± 0.10 ^c^
C22:2 n-6	-	0.03 ± 0.01 ^a^	-	0.04 ± 0.01 ^a^
C22:6 n-3	2.36 ± 0.08 ^a^	3.61 ± 0.08 ^b^	3.08 ± 0.19 ^c^	4.02 ± 0.06 ^d^
***PUFAs***	***15.43 ± 0.56*** ^a^	***18.39 ± 0.55*** ^b^	***13.79 ± 0.76*** ^a,c^	***19.17 ± 0.37*** ^b,d^
***n6/n3***	***0.84***	***0.89***	***0.69***	***0.80***

^a–c^: Different superscript letters in the same row indicate significantly different values (*p* < 0.05 by post hoc Tukey’s HSD test). Same superscript letters in the same row indicate not significantly different values (*p* > 0.05 by post hoc Tukey’s HSD test).

**Table 2:** Elemental signatures of male and female jellyfishes’ bell and oral arms revealed by Inductively Coupled Plasma-Mass Spectrometry (ICP-MS). Contents of major elements (mg 100 g^−1^) and trace elements (µg 100 g^−1^) are expressed as mean ± SD (*n* = 3) on a dw basis.

	Female		Male	
	Bell	Oral Arms	Bell	Oral Arms
*Major elements (mg 100 g^−1^)*				
**Na**	6544 ± 263 ^a^	3887 ± 133 ^b^	8079 ± 318 ^c^	3740 ± 155 ^b,d^
**Mg**	692 ± 36 ^a^	427 ± 37 ^b^	650 ± 26 ^a,c^	440 ± 20 ^b,d^
**K**	196 ± 13 ^a^	126 ± 14 ^b^	229 ± 21 ^a,c^	143 ± 14 ^b,d^
**Ca**	215 ± 22 ^a^	143 ± 13 ^b^	236 ± 12 ^a,c^	133 ± 10 ^b,d^
*Essential trace elements (µg 100 g^−1^)*				
**Fe**	1465 ± 133 ^a^	854 ± 72 ^b^	1309 ± 140 ^a,c^	1085 ± 114 ^b,d^
**Cu**	699 ± 96 ^a^	1095 ± 97 ^b^	555 ± 83 ^a,c^	1424 ± 180 ^d^
**Zn**	570 ± 67 ^a^	939 ± 62 ^b^	695 ± 42 ^a,c^	1106 ± 131 ^b,d^
**Mn**	49.7 ± 5.99 ^a^	77.8 ± 16 ^b^	146 ± 20 ^c^	212 ± 37 ^d^
**Se**	46.2 ± 4.8 ^a^	100 ± 12 ^b^	31.2 ± 3.6 ^a,c^	115 ± 39 ^b,d^
*Nonessential trace elements (µg 100 g^−1^)*				
**Cr**	401 ± 30 ^a^	690 ± 45 ^b^	573 ± 58 ^c^	668 ± 93 ^d^
**Ni**	142 ± 12 ^a^	207 ± 19 ^b^	178 ± 14 ^b,c^	215 ± 9.5 ^b,d^
**As**	412 ± 66 ^a^	631 ± 94 ^b^	528 ± 48 ^b^	663 ± 90 ^b,c^
**Cd**	42.6 ± 4.6 ^a^	40.4 ± 2.8 ^a^	46.2 ± 7.02 ^a^	51.7 ± 10.6 ^a^
**Pb**	140 ± 19 ^a^	154 ± 15 ^a^	117 ± 12 ^a^	132 ± 16 ^a^

^a–c^: Different superscript letters in the same row indicate significantly different values (*p* < 0.05 by post hoc Tukey’s HSD test). Same superscript letters in the same row indicate not significantly different values (*p* > 0.05 by post hoc Tukey’s HSD test).

**Table 3:** Sample pools considered for the present study.

Collected Specimens	Pool	*n*
200 males	Bells	200
	Oral arms	200
200 females	Bells	200
	Oral arms	200

**Table 4:** ICP-MS operating conditions applied to elemental analysis.

Parameter	Value
Forward power	1550 W
Plasma gas flow rate (Ar)	14 L min^−1^
Auxiliary gas flow rate (Ar)	0.89 L min^−1^
Carrier gas flow rate (Ar)	0.91 L min^−1^
Collision gas flow rate (He)	4.5 mL min^−1^
Spray chamber temperature	2.70 °C
Sample uptake/wash time	45 s
Injection volume	200 μL
Sample introduction flow rate	0.93 mL min^−1^
Scan mode	Full scan
Dwell time	Optimized for each analyte

## Supplementary Materials

The following are available online at https://www.mdpi.com/1660-3397/17/3/172/s1, Table S1: Summary output of the two-way ANOVA with replication analysis performed on biometric data from *P. noctiluca.* SS: sum-of-squares; df: degrees of freedom; MS: mean squares. Table S2. Summary output of the two-way ANOVA with replication analysis performed on gross energy contents from *P. noctiluca.* SS: sum-of-squares; df: degrees of freedom; MS: mean squares. Table S3. Summary output of the two-way ANOVA with replication analysis performed on crude protein contents derived from *P. noctiluca*. SS: sum-of-squares; df: degrees of freedom; MS: mean squares. Table S4. Summary output of the two-way ANOVA with replication analysis performed on total polyphenol levels obtained from *P. noctiluca.* SS: sum-of-squares; df: degrees of freedom; MS: mean squares. Table S5: Performance of the ICP-MS method in terms of linearity, LOD, LOQ, intra- and interday repeatability (*n* = 3), and accuracy.
