# Supplementary material for: A Multi-screening Evaluation of the Nutritional and Nutraceutical Potential of the Mediterranean Jellyfish Pelagia noctiluca
Source: Mar Drugs. 2019 Mar 17;17(3):172. doi: 10.3390/md17030172 (PMC6470882; doi:10.3390/md17030172)
Supplement: Supplementary file 1 [file marinedrugs-17-00172-s001.pdf]

Table S1. Summary output of the two-way ANOVA with replication analysis performed on biometric data from *P. noctiluca.* SS: sum-of-squares; df: degrees of freedom; MS: mean squares.

Source of variation	*SS*	*df*	*MS*	*F-statistics*	*P-value*	*F critical value*
**Dependent variable: weight**						
**Independent variable: Sampling month**	2767.414533	3	922.4715111	246.7524004	1.32748E-13	3.238871522
**Independent variable: Sex**	0.000416667	1	0.000416667	0.000111454	0.991707249	4.493998418
**Interaction**	82.83018333	3	27.61006111	7.385430088	0.002526234	3.238871522
**Within**	59.8152	16	3.73845			
**Total**	2910.060333	23				
**Dependent variable: lenght**						
**Independent variable: Sampling month**	25.93311667	3	8.644372222	8.51910587	0.001306733	3.238871522
**Independent variable: Sex**	33.04106667	1	33.04106667	32.56226568	3.24525E-05	4.493998418
**Interaction**	27.20533333	3	9.068444444	8.937032824	0.001038041	3.238871522
**Within**	16.23526667	16	1.014704167			
**Total**	102.4147833	23				
**Dependent variable: bell diameter**						
**Independent variable: Sampling month**	22.17894583	3	7.392981944	17.10315655	3.02209E-05	3.238871522
**Independent variable: Sex**	0.182004167	1	0.182004167	0.421054154	0.525616673	4.493998418
**Interaction**	6.431579167	3	2.143859722	4.959672392	0.012726122	3.238871522
**Within**	6.916133333	16	0.432258333			
**Total**	35.7086625	23				

Table S2. Summary output of the two-way ANOVA with replication analysis performed on gross energy contents from *P. noctiluca.* SS: sum-of-squares; df: degrees of freedom; MS: mean squares.

Source of variation	*SS*	*df*	*MS*	*F-statistics*	*P-value*	*F critical value*
**Indipendent variable: sex**	61152.95968	1	61152.96	203.2512	5.71E-07	5.317655
**Indipendent variable: anatomical part**	316250.6578	1	316250.7	1051.107	8.93E-10	5.317655
**Interaction**	43022.82204	1	43022.82	142.9929	2.2E-06	5.317655
**Within**	2406.990141	8	300.8738			
**Total**	422833.4297	11				

Table S3. Summary output of the two-way ANOVA with replication analysis performed on crude protein contents derived from *P. noctiluca*. SS: sum-of-squares; df: degrees of freedom; MS: mean squares.

Source of variation	*SS*	*df*	*MS*	*F-statistics*	*P-value*	*F critical value*
**Indipendent variable: sex**	0.980408	1	0.980408	10.66724	0.011423	5.317655
**Indipendent variable: anatomical part**	8.687008	1	8.687008	94.51818	1.05E-05	5.317655
**Interaction**	0.008008	1	0.008008	0.087134	0.775369	5.317655
**Within**	0.735267	8	0.091908			
**Total**	10.41069	11				

Table S4. Summary output of the two-way ANOVA with replication analysis performed on total polyphenol levels obtained from *P. noctiluca.* SS: sum-of-squares; df: degrees of freedom; MS: mean squares.

Source of variation	*SS*	*df*	*MS*	*F-statistics*	*P-value*	*F critical value*
**Indipendent variable: sex**	291057	1	291057,0269	2653,929436	2,23334E-11	5,317655063
**Indipendent variable: anatomical part**	2434603	1	2434602,63	22199,30456	4,60572E-15	5,317655063
**Interaction**	18073,47	1	18073,4692	164,7983298	1,28016E-06	5,317655063
**Within**	877,3618	8	109,6702207			
**Total**	2744610	11				

Table S5. Performance of the ICP-MS method in terms of linearity, LOD, LOQ, intra- and interday repeatability (n=3), and accuracy.

**Element**	**a intercept**	**b slope**	R^2	**LOD (ng g^{-1})**	**LOQ (ng g^{-1})**	**Precision (RSD%) Intraday**	**Interday**	**Recovery (%)**
**^{23}Na**	$6.77 \cdot 10^5$	$3.78 \cdot 10^7$	0.99971	0.21	0.69	2.84	3.22	100.29
**^{24}Mg**	$7.37 \cdot 10^7$	$4.12 \cdot 10^8$	0.99428	0.15	0.50	3.36	4.06	96.73
**^{39}K**	$1.41 \cdot 10^7$	$4.82 \cdot 10^8$	0.99863	0.25	0.82	2.10	3.42	95.65
**^{44}Ca**	$4.73 \cdot 10^6$	$1.97 \cdot 10^7$	0.99454	0.14	0.47	5.53	6.33	97.77
**^{52}Cr**	$1.29 \cdot 10^7$	$2.87 \cdot 10^7$	0.99895	0.018	0.060	4.73	6.20	103.31
**^{55}Mn**	$8.41 \cdot 10^7$	$1.40 \cdot 10^7$	0.99993	0.12	0.40	4.25	4.43	99.17
**^{56}Fe**	$4.02 \cdot 10^6$	$3.89 \cdot 10^8$	0.99740	0.15	0.51	6.53	7.09	90.53

**^{60}Ni**	$6.51 \cdot 10^{6}$	$9.95 \cdot 10^{9}$	0.99887	0.010	0.033	4.11	4.74	94.67
**^{63}Cu**	$1.99 \cdot 10^{9}$	$2.76 \cdot 10^{10}$	0.99991	0.14	0.48	6.82	7.11	102.89
**^{66}Zn**	$2.27 \cdot 10^{8}$	$3.51 \cdot 10^{9}$	0.99982	0.22	0.73	2.02	3.15	99.67
**^{75}As**	$1.64 \cdot 10^{8}$	$2.96 \cdot 10^{9}$	0.99917	0.016	0.053	3.18	4.17	102.37
**^{78}Se**	$1.95 \cdot 10^{7}$	$2.14 \cdot 10^{10}$	0.99940	0.015	0.050	4.56	5.34	93.49
**^{111}Cd**	$5.97 \cdot 10^{5}$	$1.45 \cdot 10^{7}$	0.99988	0.012	0.040	6.32	8.52	99.18
**^{208}Pb**	$4.84 \cdot 10^{8}$	$2.51 \cdot 10^{8}$	0.99999	0.016	0.053	5.47	6.96	97.89
